# Infection of Brindley sacral anterior root stimulator by *Pseudomonas aeruginosa *requiring removal of the implant: long-term deleterious effects on bowel and urinary bladder function in a spinal cord injury patient with tetraplegia: a case report

**DOI:** 10.1186/1757-1626-2-9364

**Published:** 2009-12-21

**Authors:** Subramanian Vaidyanathan, Bakul M Soni, Tun Oo, Peter L Hughes, Paul Mansour, Gurpreet Singh

**Affiliations:** 1 Spinal Injuries Unit, District General Hospital, Town Lane, Southport PR8 6PN, UK; 2 Department of Radiology, District General Hospital, Southport PR8 6PN, UK; 3 Department of Cellular Pathology, District General Hospital, Southport PR8 6PN, UK; 4 Department of Urology, District General Hospital, Southport PR8 6PN, UK

## Abstract

**Introduction:**

We report infection of Brindley sacral anterior root stimulator in a spinal cord injury patient, who ultimately required removal of the implant. The consequences of failed implantation were severe constipation, and loss of reflex penile erection and bladder emptying.

**Case presentation:**

A male patient, born in 1973, fell off the balcony while on holidays in Crete in 1993 and developed complete tetraplegia at C-5 level. In 1996, deafferentation of sacral nerve roots 2, 3 and 4 were carried out bilaterally. Brindley sacral anterior root stimulator was implanted. On eleventh post-operative day, blood stained fluid came out of sacral wound. Microbiology of exudates showed growth of *Pseudomonas aeruginosa*, sensitive to gentamicin. As discharge of serosanguinous fluid persisted, sacral wound was explored. In March 1997, induration and craggy swelling were noted at the site of receiver. There was discharge from the surgical wound in the back. Wound swab grew *Pseudomonas aeruginosa*. The receiver was taken out. Cables were retrieved and tunnelled in left flank. Laminectomy wound was left open. In May 1997, cables were removed from left flank through the laminectomy wound. Grommet was sliced down as much as possible without producing leak of cerebrospinal fluid. Histoacryl glue was used over the truncated grommet as a sealing agent. Microbiology of end of S-2 and S-3 cables showed growth of *Pseudomonas aeruginosa*, which was sensitive to gentamicin. End of S-4 cable showed scanty growth of *Pseudomonas aeruginosa *and *Klebsiella aerogenes*. Review of this patient in January 1999 revealed presence of sinuses in dorsal wound exuding purulent material. The wound was explored; grommet and electrodes were removed. The consequences of failed implantation were severe constipation and loss of reflex penile erection and bladder emptying. This patient had to spend increasing amount of time for bowels management. Faecal incontinence limited his mobility. The problem with his bowels was affecting his confidence in doing anything, as the slightest movement could cause his bowels to work. The inconvenience and embarrassment of a bowel accident caused distress to the patient and to his mother.

**Conclusion:**

This case illustrates that bacterial infection is a major problem in spinal cord injury patients who undergo implantation of medical devices. Further, this case underlines the need for honest discussion with spinal cord injury patients about possible complications of implantation of sacral anterior root stimulator and long-term consequences of an unsuccessful operation.

## Introduction

Persons with spinal cord injury appear to be more susceptible to get bacterial infection than able-bodied individuals. *Pseudomonas aeruginosa *was cultured from one or more body sites (urethra, perineum, or rectum) in 65% of men and 18% of women with spinal cord injury [1]. Drainage bags on the beds were frequently colonized with *Pseudomonas aeruginosa *(73%). A review of literature shows that spinal cord injury recipients of penile prostheses are at increased risk for periprosthetic infection and erosion. Montague and Lakin [2] recommended that men with spinal cord injury should be encouraged to consider alternative treatments for erectile dysfunction such as vacuum constrictive devices or intracavernous injection therapy instead of implantation of penile prosthesis. Further, when men with spinal cord injury elect penile prosthesis implantation, they should be informed of the increased risks for infection and erosion. We report infection of Brindley sacral anterior root stimulator in a spinal cord injury patient, who ultimately required removal of the implant. The consequences of failed implantation were severe constipation and loss of both reflex penile erection and bladder emptying. The deleterious effects on bowel function were frustrating, and compromised the quality of life in this young tetraplegic subject.

## Case presentation

A male patient, who was born in 1973, fell off the balcony while on holidays in Crete in 1993. He sustained fractures of the bodies of C-4, C-5 and C-6 vertebrae and developed complete tetraplegia at C-5 level. Suprapubic cystostomy was performed on 25 August 1993. The suprapubic catheter came out on 03 October 1993. Therefore, per urethral catheter was inserted. In December 1993, the balloon of Foley catheter got burst and catheter fell off. Ultrasound of urinary bladder showed a reflective calculus with acoustic attenuation measuring 1.9 cm. Cystoscopy and electrohydraulic lithotripsy of vesical calculus were carried out on 14 January 1994. In August 1994, this patient attended spinal unit with history of frequent bypassing of urethral catheter. X-ray of urinary bladder revealed vesical calculus. On 02 September 1994, cystoscopy and electrohydraulic lithotripsy were carried out. In 1995, there was recurrence of stones in urinary bladder. Electrohydraulic lithotripsy of vesical calculi was performed on 17 November 1995.

On 27 September 1996, deafferentation of sacral nerve roots 2, 3 and 4 were carried out bilaterally. Brindley sacral anterior root stimulator was implanted. Standard closure of dura was performed. 120 mg of gentamicin and 1.5 gram of cefuroxime were administered just prior to surgery. On 02 October 1996, sacral stimulator was checked. When S-3 was stimulated, erection of penis occurred and this stopped flow of urine. Stimulation of S-2 produced good erection. Stimulation of S-3 and S-4 resulted in a rise of intravesical pressure to 50 cm of water. On 08 October 1996, blood stained fluid came out of sacral wound. Microbiology of exudates showed growth of *Pseudomonas aeruginosa*, sensitive to gentamicin. As discharge of serosanguinous fluid persisted, sacral wound was explored on 22 October 1996. There was probably a minor leak of cerebrospinal fluid just above the grommet. Several sutures were placed around the grommet; several layers of tissue were fixed as secondary collar and a minute amount of Histoacryl was used. Wound was closed meticulously in several layers. Intrathecal drain was kept. The patient was nursed in prone position after surgery. On 08 November 1996, there was a sudden release of bloodstained cloudy fluid from the dorsal wound. This patient was prescribed cefotaxime one gram every eight hours and, metronidazole 500 mg every eight hours. Microbiology of specimen taken from lumbar wound on 20 November 1996, showed heavy growth of *Pseudomonas aeruginosa*; this organism was sensitive to gentamicin.

**Figure 1:**
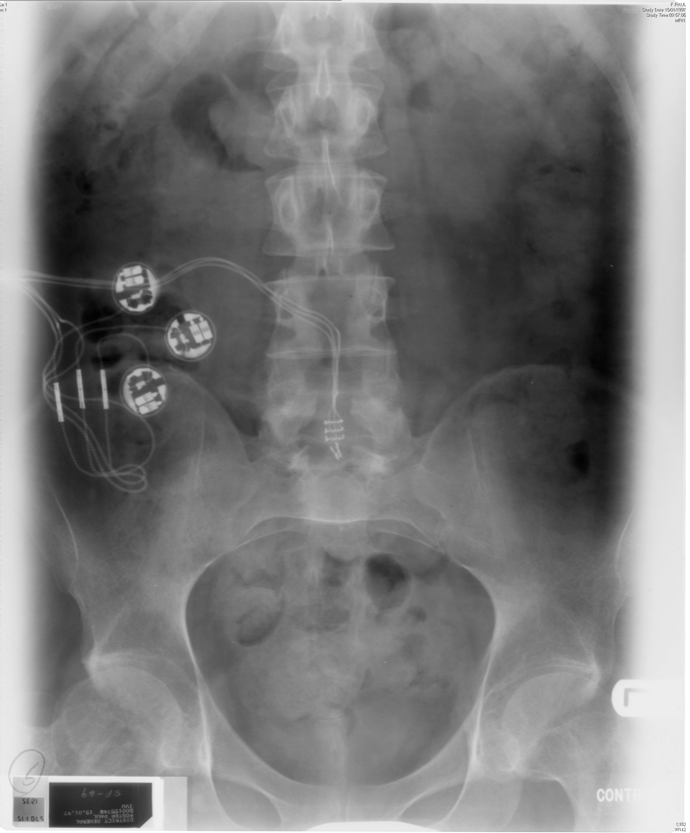
**X-ray of abdomen, taken on 15 January 1997, showed all components of Brindley stimulator (receiver block, cables, and electrodes) in place**.

**Figure 2:**
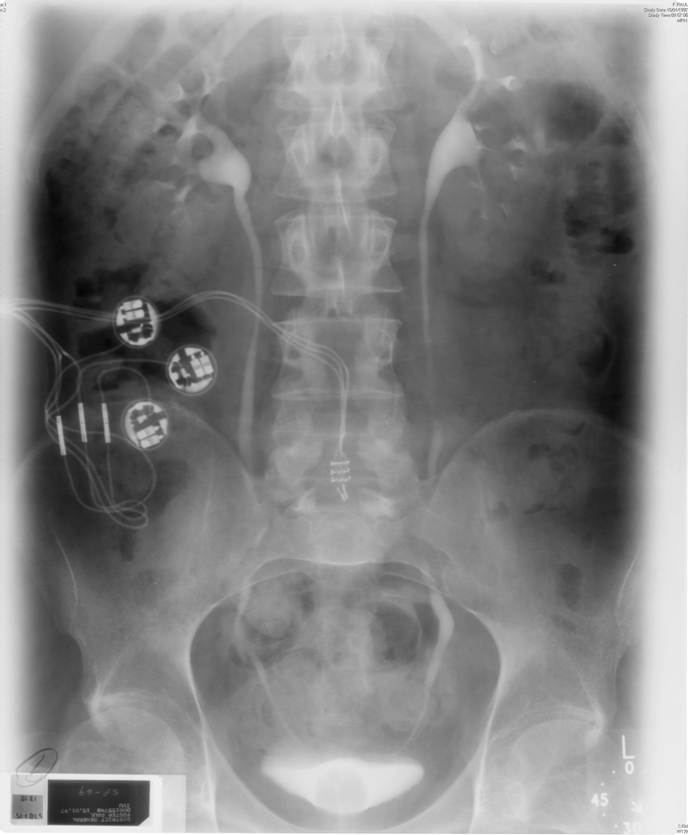
**Intravenous urography, which was performed on 15 January 1997 (35 minutes film), showed normal kidneys, ureters and bladder**. Balloon of Foley catheter was seen in situ.

Intravenous urography, performed on 15 January 1997, showed normal appearances of kidneys. (Figures 1 and 2)

**Figure 3:**
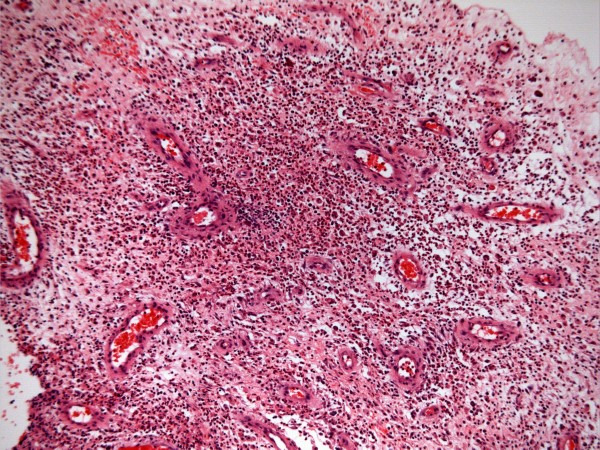
**Ulcerated, inflamed granulation tissue from abdominal wound, with numerous polymorphs and vessels of varying sizes within loose connective tissue**.

**Figure 4:**
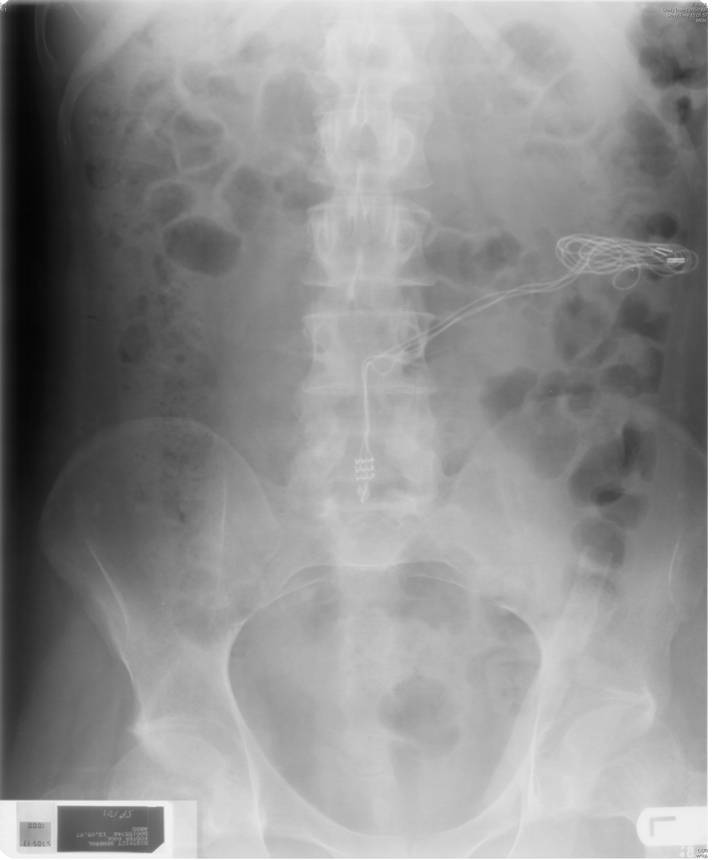
**X-ray of abdomen, taken on 13 May 1997, showed the cables tunnelled in left flank**. Receiver block had been removed. Please compare with Figure 1.

On 24 February 1997, a fluctuant swelling was noticed over dorsal midline operative scar. Aspiration yielded thick, purulent fluid. On 25 February 1997, wound swab revealed growth of *Pseudomonas aeruginosa*. On 04 March 1997, induration and craggy swelling were noted at the site of receiver. There was discharge from the surgical wound in the back. Wound swab grew *Pseudomonas aeruginosa *on 05 March 1997. On 07 March 1997, receiver site was opened. Tissue appeared infected; biopsy was taken. The receiver was taken out. Cables were retrieved; granulation tissue was present all along subcutaneous tunnel. Laminectomy wound was opened. No leak of cerebrospinal fluid was noted. Granulation tissue around entire block was cleaned; diathermy was applied. All cables were tunnelled in left flank. Gentamicin beads (23) were left along with cables. Laminectomy wound was left open. Left flank wound was closed. This patient received gentamicin 240 mg and cefuroxime intravenously. Histology showed heavily inflamed non-specific granulation tissue without frank abscess formation. There was no significant population of eosinophils to suggest an allergic reaction and no foreign material was identified. There was no evidence of neoplasia. (Figure 3) X-ray of abdomen, taken on 13 May 1997, showed the cables tunnelled in left flank. (Figure 4)

**Figure 5:**
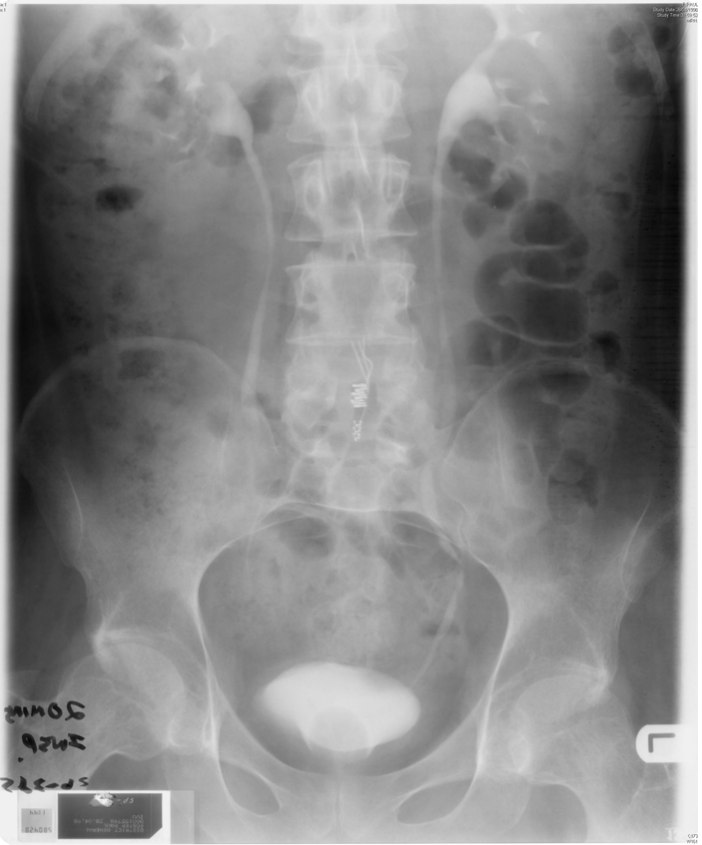
**Intravenous urography, which was performed on 28 April 1998 (20 minutes film), showed excretion of contrast by both kidneys**. The cables of Brindley stimulator, which were tunnelled in left flank, had been removed.

Microbiology of exudates from the wound grew Pseudomonas aeruginosa, which was sensitive to gentamicin and piperacillin. On 21 March 1997, this patient was prescribed piperacillin 4 grams intravenously every eight hours. On 26 March 1997, the wound in left flank was opened. Gentamicin bead chain was removed. Sacral anterior root stimulator cables were brought out and buried deeper in the flank wound. Wound was closed in layers. On 30 May 1997, cables were removed from left flank through the laminectomy wound. Grommet was sliced down as much as possible without producing leak of cerebrospinal fluid. Histoacryl glue was used over the truncated grommet as a sealing agent. The wound was closed in two layers. This patient received 340 mg of gentamicin during surgery. Microbiology of end of S-2 and S-3 cables showed growth of *Pseudomonas aeruginosa*, which was sensitive to gentamicin. End of S-4 cable showed scanty growth of *Pseudomonas aeruginosa *and *Klebsiella aerogenes*. On 04 July 1997, superficial dehiscence of the wound was noticed along with some discharge. Probing did not show any deeper extension. Since deeper extension was unlikely, it was decided not to carry out any further intervention such as removal of intradural components. Intravenous urography, performed on 28 April 1998, showed normal kidneys. (Figure 5)

**Figure 6:**
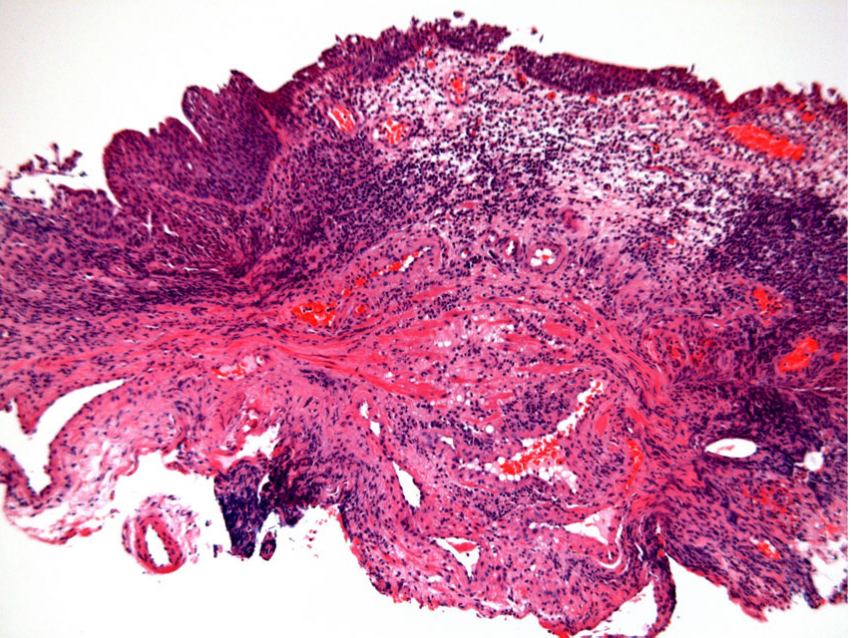
**Benign bladder biopsy (epithelium at top), showing marked inflammation predominantly beneath the epithelium but also in deeper layers of the bladder wall, and without ulceration**.

In November 1998, this patient experienced frequent blocking of urethral catheter. Flexible cystoscopy revealed hair, debris and stones in the bladder. On 20 November 1998, cystoscopy was performed; debris, hair and stones were removed. Bladder biopsy was taken. Histology revealed severe inflammation throughout, predominantly chronic (lymphocytes and plasma cells) with occasional neutrophils also. Small numbers of lymphocytes and neutrophils were present within the urothelium. (Figure 6)

This patient was reviewed on 21 January 1999. There were sinuses in dorsal wound, which were exuding purulent material. Therefore, it was decided to open the wound, clean the wound thoroughly and remove the entire system including residual cables and intradural electrodes. On 22 January 1999, the grommet, and electrodes were removed.

Bowels became troublesome. Management of bowel was unacceptable, as it required substantial amount of time. Bowel accidents caused demeaned quality of life. Since January 1999, this patient required manual evacuation twice daily. He was taking Movicol. This patient did not develop autonomic dysreflexia when he was constipated. In April 2000, this patient was admitted to spinal unit with bowel problems. This patient tried using anal plugs of large size; still he was getting bowel accidents, which caused considerable embarrassment. He had to spend increasing amount of time for attending to his bowels. He was exasperated and frustrated. Faecal incontinence limited his mobility. The problem with his bowels was affecting his confidence in doing anything, as the slightest movement could cause his bowels to work. The inconvenience and embarrassment of a bowel accident was causing distress to the patient and to his mother. In view of the desperate situation that he was in, this patient was offered the option of colostomy. In November 1999, this patient was referred to stoma nurse as colostomy was considered as an alternative bowel management route. At the present moment, this patient was reluctant to take the option of abdominal stoma for emptying his bowels.

In January 2002, this patient tried sildenafil 75 mg on eight occasions. He could not get satisfactory erection of penis. Penile erection did not last long or did not get hard enough to have sex. Even after taking 100 mg of sildenafil, this patient with tetraplegia could not achieve erection of penis. In March 2002, this patient's partner was taught to perform intra-cavernosal injection of alprostadil. A dose of 3.75 micrograms was required to be injected intra-cavernosally to produce adequate erection of penis.

**Figure 7:**
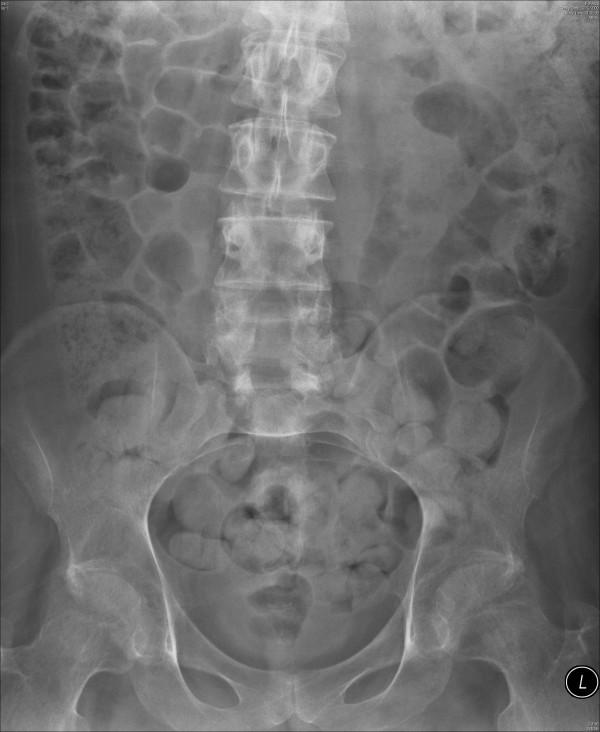
**X-ray of abdomen, taken on 26 October 2009, showed marked faecal loading through the colon**. Electrodes of Brindley stimulator, originally placed in sacral region, were no longer visible, as they had been removed. Please compare with Figure 1.

**Figure 8:**
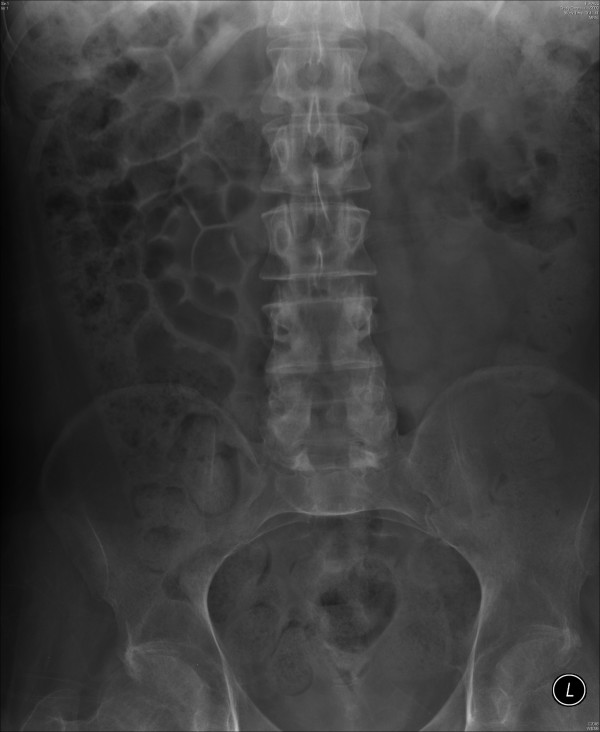
**X-ray of abdomen, taken on 11 November 2009, showed faecal loading of the colon**.

In January 2006, flexible cystoscopy revealed a calculus in bladder. On 27 January 2006, cystoscopy and electrhydraulic lithotripsy were carried out. Bowel management continued to be unsatisfactory. X-ray of abdomen, which was taken on 30 January 2006, showed marked faecal loading of the colon. At present, this patient manages his bladder by long-term indwelling urethral catheter. X-ray of abdomen, taken on 26 October 2009, showed marked faecal loading through the colon. (Figure 7) X-ray of abdomen, taken on 11 November 2009, showed faecal loading of the colon; no radio opaque calculi were seen. (Figure 8)

## Discussion

Infection still represents one of the most serious and ravaging complications associated with prosthetic devices [3]. Kutzenberger [4] reviewed 464 paraplegic patients (220 females, 244 males), who underwent sacral deafferentation and implantation of a sacral anterior root stimulator between September 1986 and December 2002. Early complications were 6 cerebrospinal fluid leaks and 5 implant infections. Late complications included receiver or cable failures and required surgical repair in 44 patients.

Antibiotic prophylaxis significantly reduces infectious complications in patients undergoing implantation of pacemakers or cardioverter-defibrillators [5]. Our patient developed infection of implant by *Pseudomonas aeruginosa*; he received Gentamicin 120 mg during induction of anaesthesia. *Pseudomonas aeruginosa*, which was isolated from the surgical wound subsequently, was sensitive to gentamicin. In hindsight, we realise that we might have been prevented infection of implant with *Pseudomonas aeruginosa *had we administered a more appropriate dose of gentamicin (5 mg per kg) instead of 120 mg.

Deafferentation of sacral nerve roots 2, 3 and 4 resulted in loss of reflex activity in bowels, urinary bladder and penis. Consequently, this patient was unable to evacuate his bowels by using stimulant laxatives such as senna or bisacodyl. Similarly, this patient was unable to empty his bladder by reflex detrusor contractions. Therefore, he required long-term indwelling urinary catheter. Inability to achieve reflex erection of penis affected the quality of his sex life. The long-term consequences of unsuccessful bladder stimulator surgery were keenly felt during activities of daily living, as he could not achieve reflex emptying of bowels. The inconvenience and embarrassment of a bowel accident was causing distress to the patient and to his mother.

## Conclusion

This case illustrates that bacterial infection is a major problem in spinal cord injury patients, who undergo implantation of medical devices. Further, this case underlines the need for honest discussion with spinal cord injury patients about possible complications of implantation of sacral anterior root stimulator and long-term consequences of an unsuccessful operation.

## Patient's perspective

I was put on the waiting list for a 'route bladder stimulator' around about March 1996 after I was told that long term indwelling catheter was not good for my health due to the increase of urinary infections and the affect it would have on my urethra, bladder and kidneys. I had also suffered from dysreflexia when my catheter blocked, I was told that this would stop after the operation which was a real benefit of having the implant that persuaded me into agreeing to the stimulator.

In September of that year I got a phone call on a Thursday asking me if I could get to the Spinal Injuries Unit for blood tests for the operation the next day, as there had been a cancellation and I was the next on the waiting list. I agreed and went and saw my consultant who told me I would be confined to be for approx 3 weeks and the operation was about 8 hours long. I had the operation the next day.

I woke up on the Saturday in the intensive care unit of Spinal Injuries Centre with a terrible headache, this was a headache like nothing I had felt in my whole life, I was on a turning bed which was quickly positioned so my head was pointing downwards and my legs were pointing upwards, this was to stop the cerebrospinal fluid from draining out of my spinal column which was the reason for my headaches. My consultant came and saw me and tested the stimulator on the second setting, which was erection and it worked. Success; this was going to improve my life. The same day I was moved out of the intensive care unit and put into a side ward due to an elderly man becoming ill.

I was in that side ward for a couple of weeks and then moved into room 10 in low dependency ward. My wound would not heal at the bottom and I was even lying prone, which is difficult for a tetraplegic patient of my level. It was now December and the stimulator had been tested unsuccessfully on my bladder and bowel but I was told that it would often take a while for these functions to work but my wound had closed up. It was Christmas Eve and 16 weeks after my initial operation I was allowed home still on an indwelling catheter.

I went home and followed the instructions that I had been given which was to sit on the toilet and use my stimulator for my bowel to work. It did not work. Mid way through January, when I got out of bed and into my chair, for the first 10 minutes or so I started to shiver and sweat; a feeling similar to when you get a urinary tract infection. This would ease off and I would feel fine until I went to bed and then the same shivering would return for approx 10 minutes or so. This occurred every day until the beginning of March when an 'egg shape' appeared under my skin at the bottom of the wound. I returned to the Spinal Unit where my consultant put a needle in my back and withdrew all the fluid of my 'egg' and admitted me to hospital.

The following 10 months I was confined to bed whilst I undertook several operations to try to correct the problem which had been discovered; that I had infection with Pseudomonas on the silicone of the stimulator. The operations included moving wires from the stimulator from one side of my body to the other side, I had the silicone covered in Betadine, Gentamicin beads placed around the infected area and numerous powerful antibiotics, but all this did not kill the infection. Finally it was decided to remove as much of the implant as possible leaving in only the bowler hat shaped of the stimulator that went into my spinal column. After this operation I was left with a chimney shape tunnel that was approx 40 mm in diameter that went from the remaining part of the stimulator to the lower part of my back, this was packed with numerous dressings, creams including seaweed etc, as they wanted it to heal from the bottom upwards. Months later it had healed but I was left with 2 tiny holes one at the top of my scar the other near the bottom approx 75 mm apart, these 2 holes connected my stimulator like a chicken wishbone shape channel as Pseudomonas were still living on the small part of the stimulator that was left in my body. A 5 ml syringe was used to pass hydrogen peroxide through the top hole and out of the bottom on a daily basis to keep the channels open so the infection could freely run out.

I had been in bed nearly a year and it was decided that I should go home and introduce the cleaning out of the channels into my daily routine and hopefully my own body's defences would fight the infection and I would heal myself. It was now early 1998.

The rest of 1998 and most of 1999 I lived with this. I looked and felt terrible; my bowels were slow and my ability to gain a reflex erection had gone. After numerous infections and tests I met with my consultant and Professor Brindley who was the bloke who had invented the stimulator. He told me that he had never taken a stimulator out of anyone before but had taken stimulators out of baboons when inventing them. I was told that is was a dangerous operation because they would have to open my spinal column up to get the infected silicone out which could easily infect my CS fluid whilst taking it out resulting in meningitis of the CS fluid which eventually would kill me. He then told me leaving the implant in would kill me anyway, so I had no option and had the operation.

The implant was successfully taken out and I healed up quickly, as I had no infection living inside me.

### Effect the stimulator has had on me

#### 1. Bowels

##### Before Implant

I would take 15-20 mm Senna three evenings a week and I would have 5 ml Bisacodyl solution the following morning. After an hour I would have finished emptying my bowels.

##### After Implant

My bowels started to slow down virtually straight after the implant was put in. I got up to taking 40 ml Senna granules daily and Bisacodyl, which did not work because nerves had been cut. I then took Movicol, which did not work until my motion was nearly fluid. I daily had accidents and even used plugs (like tampons) to try to stop me having accidents. Finally, I went into the Spinal Unit to have a colostomy bag fitted, they took me off every medication and just gave me Fybogel. Now I have a bowel manual check everyday to remove a marble or two. I am constantly constipated, cannot each much bread (if any at all), pasta, pizza and other nice foods also stop my movement. But it is manageable.

#### 2. Erection

##### Before

Even with an indwelling catheter inserted I would get an erection with the slightest of playing around with my penis; I would also often wake up in the morning with an erection.

##### After

I did not realise that the stimulator would affect my ability to gain an erection. I noticed straight away that my ability to gain a reflex erection had gone. The stimulator did not work so I was put on Viagra, which worked for a few times then after gradually increasing the strength of Viagra I found that it stopped working. Then I was put on Caverject, which worked for a short time and then stopped.

I am a married young man and this has had a massive effect on not only our married life but on myself personally.

### Would I recommend a stimulator?

#### Personally I would not recommend a stimulator not only because it nearly cost me my life, but also how it affects your bowel and erection when it is not working properly

I know 4 other people who have the stimulator and 2 of them have had problems related to it whilst the other 2 have had no problems at all.

Although I do not get autonomic dysreflexia any more I feel having the stimulator has had quite the opposite effect of improving the quality of my life, which it was advertised to me that it would do.

I think my consultant whom put this route stimulator inside me is probably the best in the world and other experts in other fields of medicine I know all agree with me on this point, but I made the wrong decision taking his advice of having such an irreversible operation with so many additional negative effects.

If I was considering such an operation I would like to think I could discuss the positive and negative effects of the stimulator with other people who have undergone the operation.

## Competing interests

The authors declare that they have no competing interests.

## Authors' contributions

SV developed the concept and wrote draft. BMS was the Consultant in charge of this patient. PH reviewed medical images. PM reported histology of biopsies. All authors contributed to patient care. All authors read and approved the final manuscript.

## Consent

Written informed consent was obtained from the patient for publication of this case report and accompanying images. A copy of the written consent is available for review by the Editor-in-Chief of this journal.
